# HPLC-PDA Combined with Chemometrics for Quantitation of Active Components and Quality Assessment of Raw and Processed Fruits of *Xanthium strumarium* L.

**DOI:** 10.3390/molecules23020243

**Published:** 2018-01-25

**Authors:** Hai Jiang, Liu Yang, Xudong Xing, Meiling Yan, Xinyue Guo, Bingyou Yang, Qiuhong Wang, Haixue Kuang

**Affiliations:** 1 Key Laboratory of Chinese Materia Medica, Heilongjiang University of Chinese Medicine, Ministry of Education, Harbin 150040, China; jianghai_777@126.com (H.J.); hxk_yl@163.com (L.Y.); mrxing_xudong@126.com (X.X.); ymlymlyml418@163.com (M.Y.); m17645028606@163.com (X.G.); ybywater@163.com (B.Y.); 2 School of Traditional Chinese Medicine, Guangdong Pharmaceutical University, Guangzhou 528458, China

**Keywords:** Xanthii Fructus, multi-component quantitation, HPLC-PDA, quality assessment, chemometrics

## Abstract

As a valuable herbal medicine, the fruits of *Xanthium strumarium* L. (Xanthii Fructus) have been widely used in raw and processed forms to achieve different therapeutic effects in practice. In this study, a comprehensive strategy was proposed for evaluating the active components in 30 batches of raw and processed Xanthii Fructus (RXF and PXF) samples, based on high-performance liquid chromatography coupled with photodiode array detection (HPLC-PDA). Twelve common peaks were detected and eight compounds of caffeoylquinic acids were simultaneously quantified in RXF and PXF. All the analytes were detected with satisfactory linearity (R^2^ > 0.9991) over wide concentration ranges. Simultaneously, the chemically latent information was revealed by hierarchical cluster analysis (HCA) and principal component analysis (PCA). The results suggest that there were significant differences between RXF and PXF from different regions in terms of the content of eight caffeoylquinic acids. Potential chemical markers for XF were found during processing by chemometrics.

## 1. Introduction

Chinese herbal medicine (CHM), as a form of empirical drug system, has thousands of years of folk practice. As an important part of traditional Chinese medicinal theory, the CHM-processing system emphasizes the enhancement of biological efficacy and the reduction of toxicity through processing by different methods, such as stir-frying, cutting, soaking, boiling, steaming, stir-frying with rice wine, steaming with rice wine, etc. [1]. Although CHMs and related processed products are derived from the same herbal origin, their pharmacological effects vary greatly from each other [2,3]. CHMs have often also been used in either raw or processed forms to treat various diseases all over the world [4]. However, little attention has been paid to processed herbs, especially to the differences between raw and processed herbs. It is well-known that CHMs are a complex mixture containing different chemical constituents that are responsible for their therapeutic effects [5]. Importantly, their chemical pattern and content will be changed by processing [6,7,8]. It is unknown whether there are changes in the efficacy of CHMs caused by differences in the content of active elements or changes in the chemical composition. Therefore, the study of the active ingredients in raw and processed Chinese medicinal herbs is crucial in the effort to obtain a better understanding of the pharmacological basis of raw and processed CHMs.

*Xanthium strumarium* (composite) is a *Xanthium*-genus plant, with the ripe dried fruits of *Xanthium strumarium* L. (Xanthii Fructus) being used as a herbal medicine in China. It was recorded in Shen Nong’s *Herbal Classic*, one of the four classic Chinese medicine books. The effect of Xanthii Fructus (XF) is scattered dehumidification and the relief of a stuffy nose. Moreover, XF has been used extensively in China for the treatment of various diseases such as nasal sinusitis, headaches caused by cold winds, rheumatic arthralgia, skin pruritus and many others [9]. However, in traditional Chinese medical literature, two forms of XF are usually used in treatment. Raw XF (RXF) was mainly used for pruritus, vitiligo and many skin diseases. Processed XF (PXF) with stir-frying was applied for rheumatism, as an analgesic, and for many diseases [10]. Thus, it is necessary to discriminate the clinical practice of raw and processed XF, as the processing of CHMs is an empirical science [11]. The stir-flying method of XF has different variable parameters and different tests for this have been used in different regions or by different individuals. Usually assessed by color or scent, there are three degrees of frying in this process: stir-frying until yellow, stir-frying until scorched, and stir-frying until carbonized. However, standardization of the processing method for PXF has not yet been fully elucidated. Therefore, the standardization of RXF processing has become more important.

Based on literature and our previous phytochemical studies on Xanthii Fructus, we have revealed that it contains caffeoylquinic acid, lignans, ent-kauranoid glycosides, and so on [12,13,14,15,16,17]. Among these, caffeoylquinic acid is the major active ingredient of XF [12,18] with a wide range of biological effects, such as activities that are antioxidant [19], antibacterial [20], anticancer [21] and potentially anti-inflammatory [22,23,24]. However, the caffeoylquinic acids in XF are mostly isomers, and the separation and analysis of caffeoylquinic acids in XF remains challenging. Moreover, to the best of our knowledge, the simultaneous determination of multiple caffeoylquinic acids in RXF and PXF has not been reported. Currently, the chromatographic fingerprint technique is regarded as a useful method to control the quality of CHMs and their derivatives because this technique emphasizes the systemic characterization and evaluation of the stability of the components [25,26]. Due to complex multivariate datasets for the complicated composition of herbal medicines, minor differences between very similar chromatograms might be missed [27]. Moreover, chemometric approaches have been increasingly viewed as valuable complements to high-performance liquid chromatography (HPLC) practices, because a large number of variables can be simultaneously controlled to achieve the expected separations [28]. Accordingly, the combination of chromatogrphic analysis and chemometrics would be a powerful tool for the systemic assessment of CHMs.

In this study, a HPLC-PDA (photodiode array detection) method was first developed for the simultaneous determination of eight caffeoylquinic acid compounds including caffeic acid (CA); 3-*O*-caffeoylquinic acid (3-CQA); 4-*O*-caffeoylquinic acid (4-CQA); 5-*O*-caffeoylquinic acid (5-CQA); 1,3-*O*-di-caffeoylquinic acid (1,3-diCQA); 1,5-*O*-di-caffeoylquinic acid (1,5-diCQA); 4,5-*O*-di-caffeoylquinic acid (4,5-diCQA); and 1,3,5-*O*-tricaffeoylquinic acid (1,3,5-tirCQA). These were determined from 30 batches of XF samples, including stir-fried and raw samples, collected from different cities of China. Meanwhile, different compositions of PXF based on the previous method were evaluated. Furthermore, hierarchical cluster analysis (HCA) and principal component analysis (PCA) were applied to distinguish RXF and PXF from different regions based on potential biomarkers in order to predict the processing method of XF. Then, a comparison of the changes in the active ingredient between RXF and PXF was made to demonstrate the relationship between pharmacological efficacy and potential mechanism. Consequently, regions with better production of XF and processing methods were selected to establish a good foundation for further research.

## 2. Results and Discussion

### 2.1. Optimization of the Extraction Method

In this study, variables in the samples, such as type of solvent, extraction method and extraction time, were optimized. First, sample extraction using an ultrasonic bath was undertaken; this is used for the quantitative analysis of CHMs due to its convenience compared to refluxing extraction. The extraction efficiencies of different concentrations of methanol (30%, 50% and 70% methanol, and pure methanol) at each extraction time (15, 30 and 60 min) were tested. Ultimately, the optimal extraction method was finalized, as described in Section 3.2.2.

### 2.2. Optimization of Chromatographic Conditions 

To obtain satisfactory HPLC-PDA chromatograms with a better separation and sharper peaks, the types of mobile phase, column temperature and detection wavelength were optimized. Different compositions of the mobile phase (acetonitrile-water, methanol-water, acetonitrile-0.1% formic acid, methanol-0.1% formic acid) and different column temperatures (25 °C, 30 °C, 35 °C, 40 °C) were compared. The addition of formic acid to the mobile phase could improve the shape of the chromatographic peaks of eight components. Meanwhile, according to the 3D plots for eight caffeoylquinic acid components, the maximum adsorption wavelength was approximately 327 nm, which was chosen as the detection wavelength. The optimal chromatographic conditions used in this study are shown in Section 3.4.

### 2.3. Method Validation

The regression equation for each analyte showed good linearity (R^2^ > 0.9991) over their tested ranges, together with the limit of detection (LoD) and limit of quantitation (LoQ) values, as shown in Table 1. Results indicated that relative standard deviation (RSD) values for intra- and inter-day readings were 0.23–1.75% and 1.53–2.11%, respectively. The recoveries for eight constituents were in the range of 94.66–107.33%, which indicated that the established method was accurate enough for the determination of the eight compounds. Therefore, the method was precise, accurate and sensitive enough for simultaneous quantitative evaluation of the multiple compounds in XF and PXF.

### 2.4. High-Performance Liquid Chromatography (HPLC) Fingerprints and Quantification of the Eight Active Components 

#### 2.4.1. Establishment of HPLC Fingerprints of Raw Xanthii Fructus (RXF) and Processed XF (PXF)

**Figure 1:**
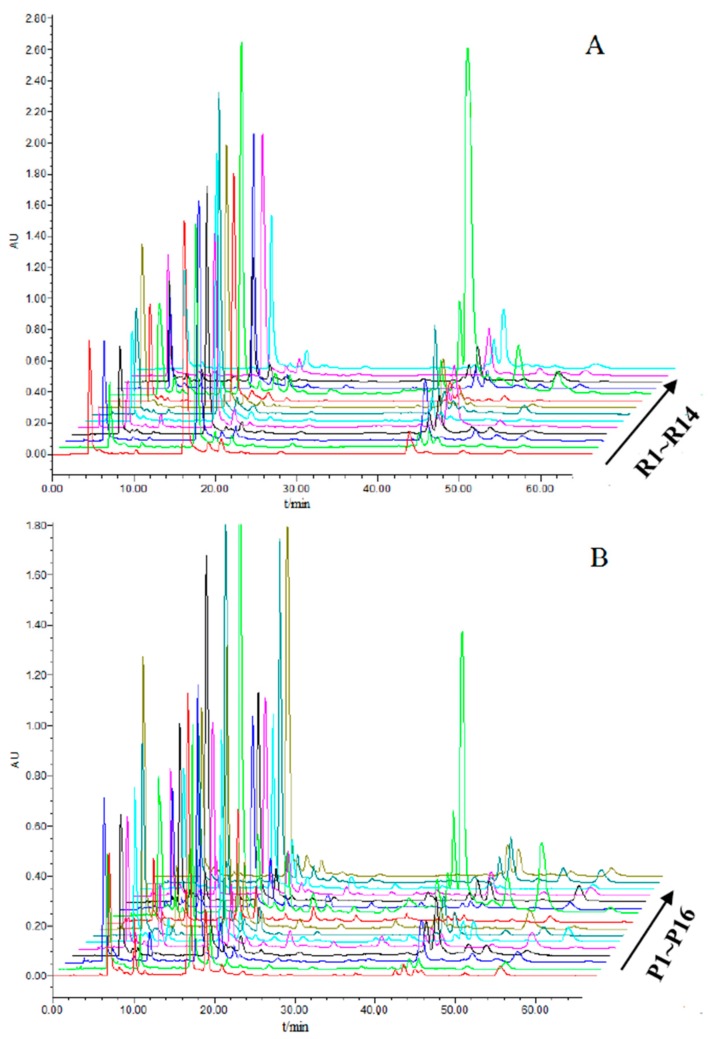
High-performance liquid chromatography–photodiode array detection (HPLC-PDA) fingerprints of (**A**) 14 batches of raw Xanthii Fructus (RXF) samples; and (**B**) 16 batches of processed XF (PXF) samples, at 327 nm.

**Figure 2:**
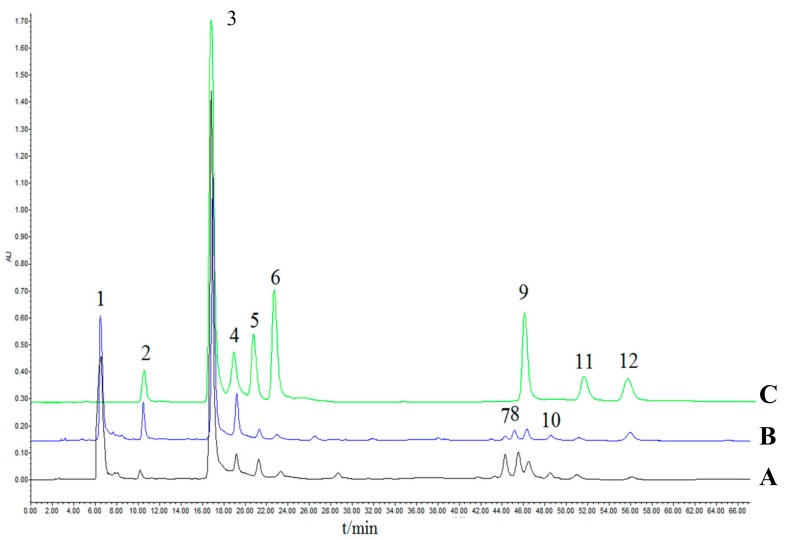
HPLC-PDA chromatograms of R9 samples: (**A**) P9 sample; (**B**) mixed standards; (**C**) at 327 nm. Peaks: (1) 1-CQA; (2) 5-CQA; (3) 3-CQA; (4) 4-CQA; (5) CA; (6) 1,3-diCQA; (7) 3,5-diCQA; (8) 1,4-diCQA; (9) 1,5-diCQA; (10) 3,4,5-triCQA; (11) 1,3,5-triCQA; (12) 4,5-diCQA.

The HPLC chromatographic fingerprints of RXF (R1-R14) are shown in Figure 1A. Similarly, 12 peaks existing in all 16 batches of PXF (P1~P16) are shown. We calculated the chromatograms dates set by all 30 samples by using the software mentioned in Section 3.7. Results showed a high degree of difference between the RXF and PXF samples with the standard fingerprint, represented in Figure 1. Simultaneously, R9 and P9 samples from the same region (Liu’an, Anhui province) were taken as examples for better comparison. As shown in Figure 2, 12 peaks existed in all 14 RXF, and 16 PXF samples with identical retention time were assigned as “common peaks” within 60 min. Furthermore, eight compounds of caffeoylquinic acid were identified among raw and processed samples by comparing the characteristic ultraviolet (UV) absorption spectra and retention time with that of the standard compounds (peaks 2, 3, 4, 5, 6, 9, 11, 12 in Figure 2). Moreover, according to previous literature [25], peaks 1, 7, 8, 10 were conjectured as 1-CQA, 3,5-diCQA, 1,4-diCQA, 3,4,5-triCQA. In the following experiments, we used the ultra-performance liquid chromatography quadrupole time of flight mass spectrometry method to confirm this.

#### 2.4.2. Quantification of Eight Caffeoylquinic Acids in Different Samples

In the present study, the eight major caffeoylquinic acids identified in the chromatographic fingerprints were quantified for RXF samples. The analyses were performed in triplicate, and results were shown in Table 2. The content of the eight caffeoylquinic acids in the 14 RXF samples varied significantly. The content of caffeoylquinic acid in the R10 sample from Dazhou, Sichuan Province, was evidently higher than that in samples from other regions. Particularly, 3-CQA had the highest content in all the RXF samples. This may be associated with the warm environmental conditions and the geographical location. Moreover, the results were also consistent with ancient literature, the Materia medica research [29]. The proposed method was subsequently applied for the quantification of eight constituents in 16 PXF samples, and the results are shown in Table 3. It was found that the content of CA, 3-CQA, 1,3-diCQA, 1,5-diCQA, 1,3,5-tirCQA decreased in some samples, and the content of 4-CQA, 5-CQA, 4,5-diCQA increased in other samples. This may be due to different processing methods for the samples. Moreover, the content of total caffeoylquinic acids in all samples was reduced. Next, the content of caffeoylquinic acids in the XF samples for different processing methods was studied.

### 2.5. Chemometric Analysis 

#### 2.5.1. Quality Evaluation by Hierarchical Cluster Analysis (HCA)

**Figure 3:**
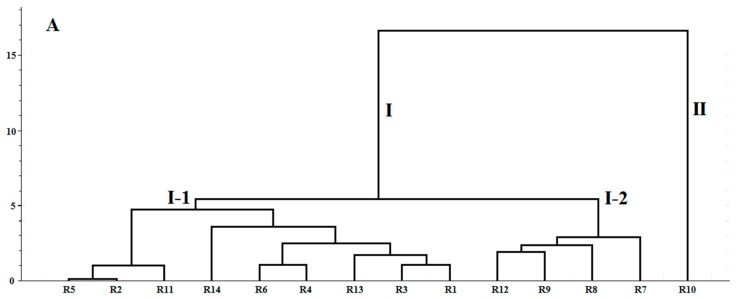

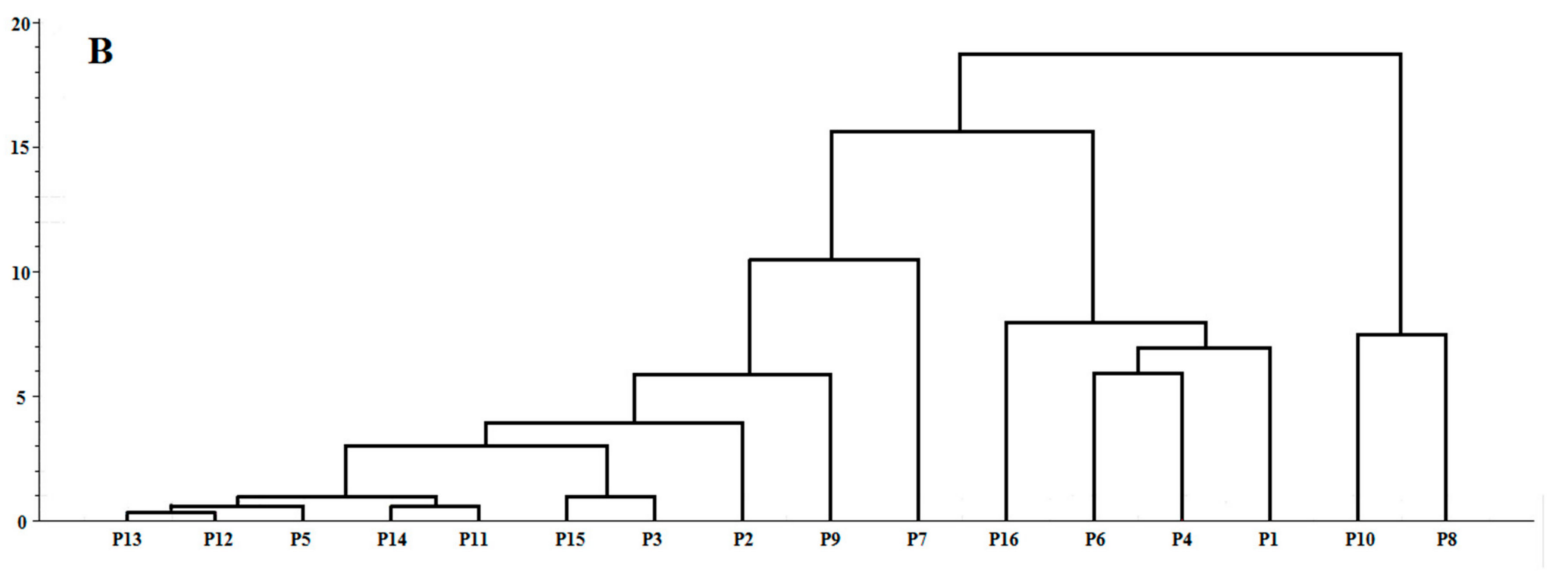
Hierarchical cluster analysis (HCA) dendrograms of: (**A**) 14 RXF samples; and (**B**) 16 PXF samples.

HCA is a useful statistical method for finding relatively homogeneous clusters of cases based on measured characteristics [30]. Samples with high similarity can be clustered into the homogenous groups. Nowadays, this method is widely used in the origin discrimination, identification and assessment of CHMs [31]. The peak areas of 12 components in the HPLC fingerprints were defined as the variables in the analysis in order to differentiate the 14 batches of RXF samples (Figure 3A).

Ward’s method was selected as a very efficient method for the analysis of variance between clusters [32]. Square Euclidean distance was selected as a measurement. The same method was applied in the 16 batches of PXF samples (Figure 3B). Two dendrograms was generated (Figure 3) to reveal the relationships among different samples. From Figure 3A, it can be seen that the 14 batches of RXF samples were divided into two main clusters: clusters I and II. Notably, the R10 sample from Dazhou, Sichuan Province, was grouped in cluster II and the other samples were in cluster I, which was consistent with the aforementioned results. Overall, according to the results of the HCA, it would be more intuitive and simple to distinguish RXF or PXF from different regions and suppliers.

#### 2.5.2. Quality Evaluation by Principal Component Analysis (PCA)

As a multivariate analysis technique, PCA can extract the dominant patterns in the matrix in terms of a complementary set of scores and loading plots in order to reduce the dimensions and the multi-indicators into a few comprehensive indicators [33]. Currently, this method is widely used in CHM composition and geographical origin analysis [34].

**Figure 4:**
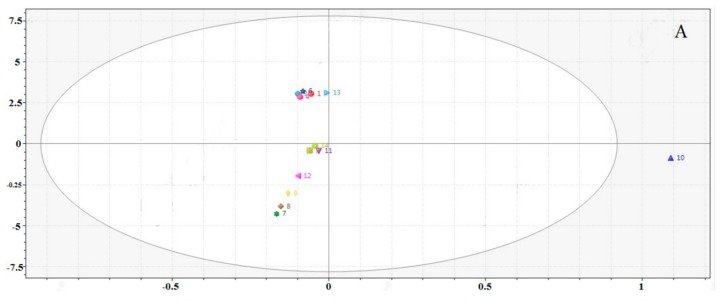

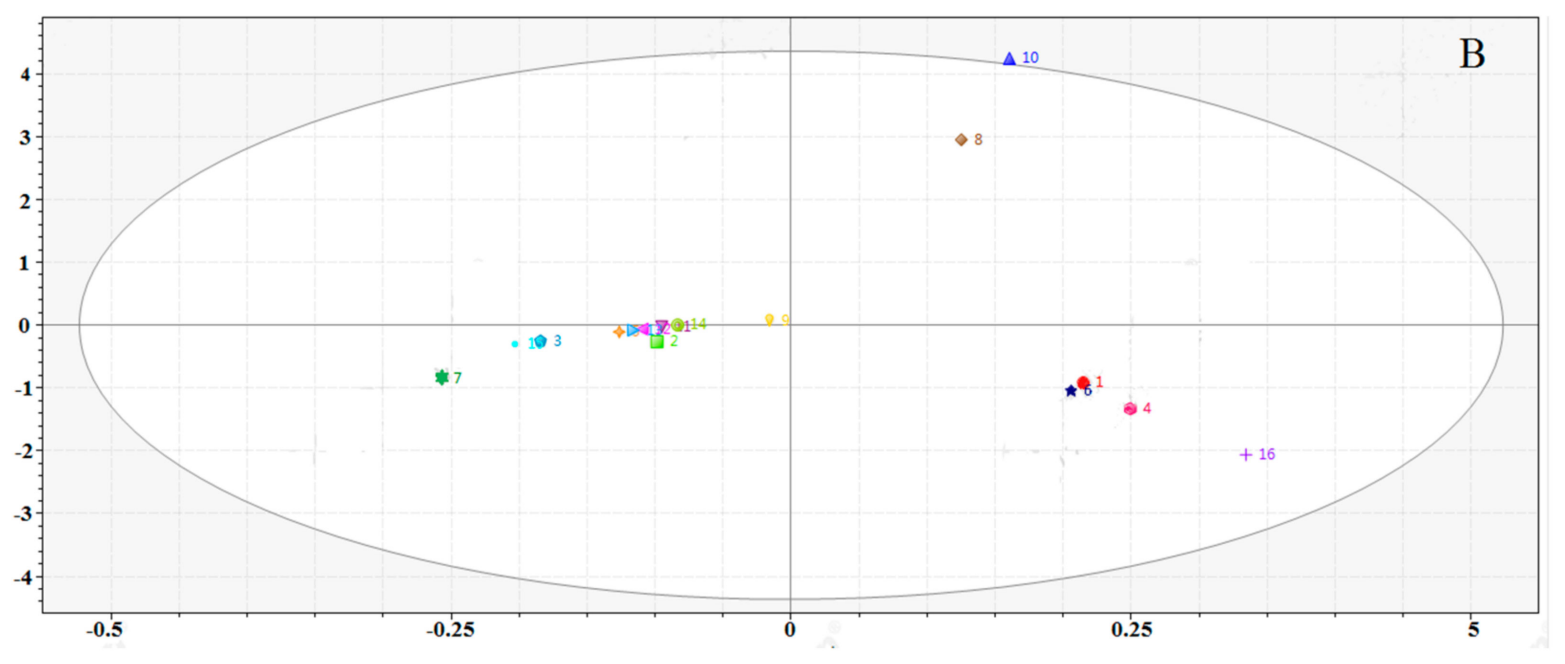
Principal component analysis (PCA) score plot of: (**A**) RXF; and (**B**) PXF.

The PCA score plot for the 14 batches of RXF samples is shown in Figure 4A, and for the 16 batches of PXF samples in Figure 4B. The R10 and P10 samples can be found from the 95% confidence intervals. In Figure 4A, all samples are clearly classified into two groups by combining PC2, respectively, which indicates that their chemical composition was obviously different. The R7 sample from Zhumadian, Henan Province; the R8 sample from Suqian, Jiangsu Province; the R9 sample from Liuan, Anhui Province; and the R12 sample from Shaoyang, Hunan Province, were classified into one group on the negative axis of PC1 and PC2. It is notable that these areas are geographically close (Figure S1), and the results revealed a pattern similar to that of HCA (Figure 3A). In particular, PXF samples from different regions or pharmacies were different in Figure 4B. Therefore, it is necessary to provide a standardized method for sample processing XF.

### 2.6. Comparison between RXF and PXF

#### 2.6.1. Comparison of Raw and Different Processed Products by Chemometric Analysis

**Figure 5:**
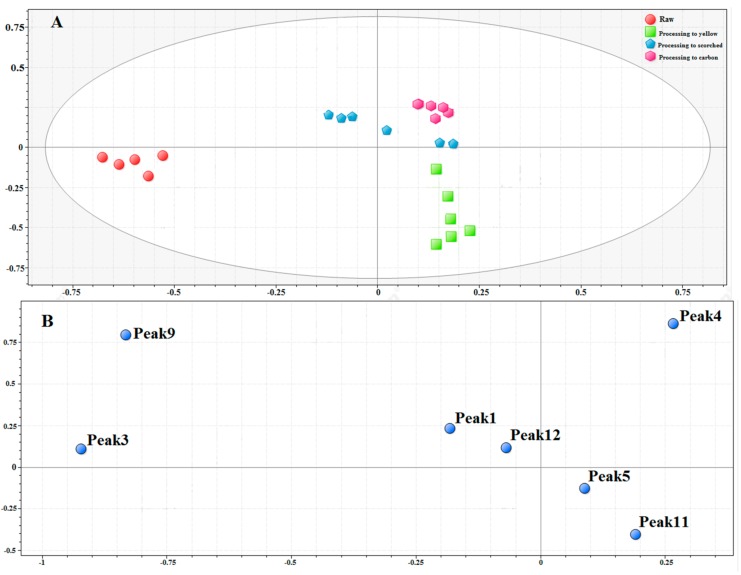
(**A**) PCA scores plot; and (**B**) loading plot for raw pieces and different processed products.

Figure 5A represents the PCA score plot, and the first principal component (PC1) accounts for 44.56% of variance whereas PC2, on the other hand, accounts for 32.91%. The final PCA score obtained using the raw pieces of XF demonstrated clear classification trends among the raw and differentially stir-fried XF samples, with all the observations falling within the 95% confidence intervals. This strong classification pattern underscored the differences between the raw and different processed products.

Furthermore, the corresponding PCA loading plot was further analyzed to find out the potential discrimination markers. Results of the PCA loading plot in Figure 5B indicate that the components of 3-CQA (peak 3), 4-CQA (peak 4), and 1,5-diCQA (peak 9) had a relatively significant influence on the difference between raw and different processed products. These components may be potential chemical markers for XF during processing.

#### 2.6.2. Quality Assessment of RXF and PXF

**Figure 6:**
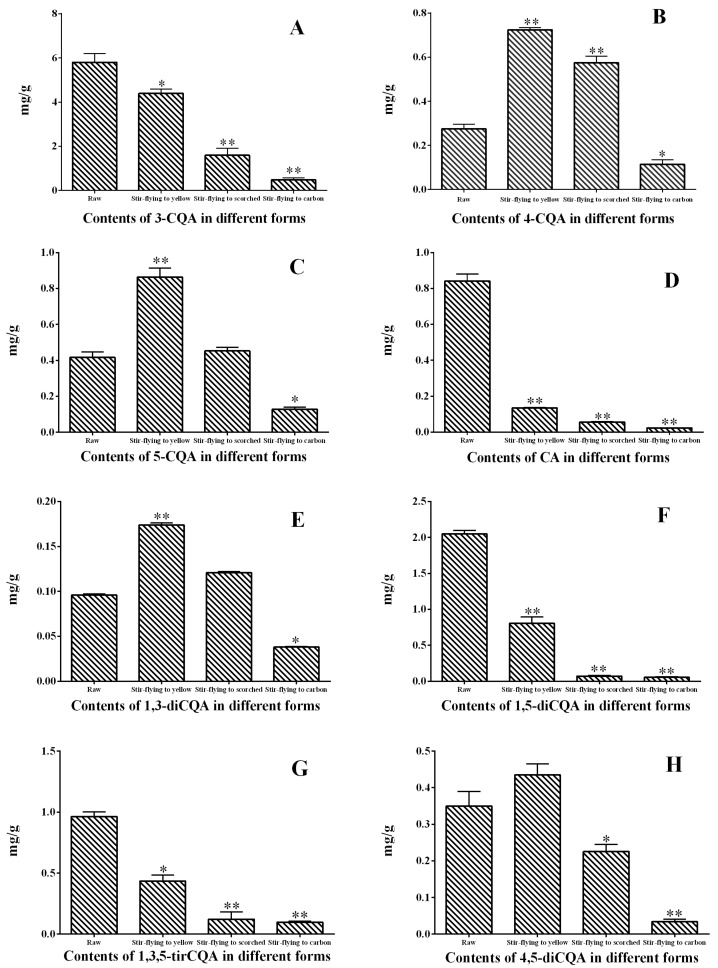
Content (mg/g) of eight components in different forms of XF samples. Data are given as mean ± SD (n = 6). Compared with raw samples, * *p* < 0.05, ** *p* < 0.01.

In order to observe the actual concentration differences of these distinct processing methods for raw XF samples, the previous HPLC-PDA method was developed for the simultaneous determination of the eight caffeoylquinic acids. Quantitative results are summarized in Figure 6. The changes in content of components during the progress of processing can be easily identified. A decreasing pattern of CA, 4-CQA, 1,5-diCQA, 1,3,5-tirCQA can be seen in the column chart (Figure 6). In particular, the content of CA and 1,5-diCQA decreased dramatically in stir-fried samples to yellow, which may be related to its thermal sensitivity. Moreover, it was found that the content of 4-CQA, 5-CQA, 1,3-diCQA and 4,5-diCQA first increased in stir-fried samples to yellow, and then decreased with the degree of processing. To the best of our knowledge, the reason for this has not been elucidated. Importantly, all the results have shown the chemical changes during the stir-frying process. Consequently, the processing method for Chinese herbal medicines must be standardized in order to ensure its quality.

Until now, few methods have been suitable for the multi-component analysis of XF samples. This study will fill the gap by providing a convenient means for this in future research on XF and its products using different processing methods.

## 3. Materials and Methods

### 3.1. Chemicals and Reagents

Methanol (HPLC grade) was obtained from Thermo Fisher Scientific (Pittsburgh, PA, USA). Formic acid was bought from Dimka Pure (Richmond Hill, NY, USA). Purified water was purchased from the Hangzhou Wahaha Group (Hangzhou, China). Others reagents and chemicals were all of analytical grade. The reference standards of CA, 3-CQA, 4-CQA, 5-CQA, 1,3-diCQA, 1,5-diCQA, 4,5-diCQA, 1,3,5-tirCQA were purchased from Chengdu Must Bio-technology Co.; Ltd. (Chengdu, China). The chemical structures of the eight reference compounds were determined to be higher than 98%.

### 3.2. Plant Materials and Sample Preparation

#### 3.2.1. Plant Materials 

Thirty batches of raw and stir-fried processing samples were collected from different regions of China (Table 2 and Table 3). All the raw samples were collected locally and the processed samples were purchased at a local pharmacy. Then, 14 batches of raw samples (R1–R14) were identified as the dried fruits of *Xanthium strumarium* L. by Prof. Zhenyue Wang (Heilongjiang University of Chinese Medicine, Harbin, China). All samples were dried to constant weight at 25 °C.

#### 3.2.2. Sample Preparation

An aliquot of 1.0 g dried powder (through a 60-mesh sieve) was accurately weighed and transferred into a 25-mL Erlenmeyer flask. After 10 mL of 50% methanol was added, the sample was extracted by ultrasonication for 60 min at room temperature. The lost weight was then made up. Finally, the supernatant of the extract was filtered through a 0.22-μm microporous membrane, and 10 μL aliquot of the filtrate was injected for HPLC-PDA analysis.

### 3.3. Standard Solution Preparation

The mixed-stock solution was prepared by dissolving each standard in methanol to obtain 2.24 mg/mL of CA; 2.16 mg/mL of 3-CQA; 2.15 mg/mL of 4-CQA; 1.31 mg/mL of 5-CQA; 2.88 mg/mL of 1,3-diCQA; 2.85 mg/mL of 1,5-diCQA; 2.19 mg/mL of 4,5-diCQA; and 2.34 mg/mL of 1,3,5-triCQA. The working standard solutions were freshly prepared by diluting suitable amounts of the above-prepared stock solution with methanol. All the solutions were stored in a refrigerator at 4 °C before analysis.

### 3.4. Chromatographic Conditions

All chromatographic analyses were performed on a Waters e2695 (Waters, Milford, MD, USA) system equipped with a PDA detector, which was controlled by the Empower 3.0 workstation. The mobile phase consisted of 0.1% formic acid (A), and methanol (B). The eight analytes were separated on a Diamonsil C-18 column (250 × 4.6 mm, 2.5 μm, Dikma, Lake Forest, CA, USA). The gradient elution program was conducted as follows: 0–15.0 min, 15–25% B; 15.0–30.0 min, 25–28% B; 30.0–33.0 min, 28–37% B; 30.0–60.0 min, 37% B. The flow rate was set at 1.0 mL/min with the injection volume of 10 μL, and the column temperature was maintained at 25 °C. The wavelength of 327 nm was selected for qualitative and quantitative analysis on the basis of the maximum absorption for all the constituents.

### 3.5. Method Validation

The mixed standard solution was diluted with methanol to yield a series of standard solutions at appropriate concentrations in order to construct the calibration curves. Limits of detection (LoDs) and quantification (LoQs) were determined with signal-to-noise (S/N) ratios of 3 and 10, respectively.

The intra-day precision of the developed method was determined by repeated injection of the sample solution six times within one day. For the inter-day test, the samples were examined in duplicates for a consecutive three days. The relative standard deviations (RSDs) were calculated for the assessment of precision. Meanwhile, an analysis of repeatability and recovery for each analyte was performed. The recovery (%) of the analytical method was evaluated by adding a known amount of the reference standards at three different concentration levels (80%, 100% and 120%) to the RXF or PXF samples.

### 3.6. Comparison between RXF and Different Processed XF Samples

**Figure 7:**
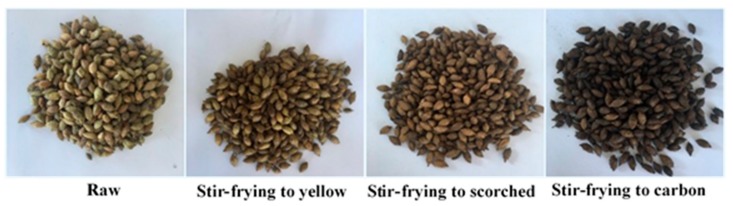
Content (mg/g) of eight components in different forms of XF samples. Data are given as mean ± SD (n = 6).

Raw pieces of XF were selected for processing research (Bozhou herbal medicine market, Anhui Province, China). The same batch of RXF was stir-fried until yellow, scorched and carbonized, respectively, according to the procedures documented in the *Chinese Pharmacopoeia* (2015 edition) [9] with different forms shown in Figure 7. Sample preparation was the same as the aforementioned procedure. For each sample, six individual extractions were performed on the raw and processed samples to generate each of the raw and processed extracts.

### 3.7. Data Analysis

All the experiments were performed at least in triplicate with constant results. Data were expressed as the mean ± RSD. Differences among groups were considered significant at *p* < 0.05. The HCA and PCA of raw or processed samples were employed for qualitative analysis using both SPSS 20.0 (IBM, Armonk, NY, USA) and SIMCA-P 13.0 software (Umetrics, San Jose, CA, USA).

## 4. Conclusions

In summary, a new HPLC-PDA method and fingerprinting analysis in conjunction with chemometric analysis was established and applied for raw and processed XF samples obtained from different locations in China. The combination of HPLC fingerprinting analysis and quantification of active ingredients with chemometric analysis provided an effective approach for discrimination and quality evaluation of XF samples from different regions. The results demonstrated the differences between XF samples from different origins and between samples from different processing methods. Importantly, the contents of caffeoylquinic acids in RXF and PXF exhibited significant variation. Therefore, these results are very important for the development of the quality control of traditional Chinese medicine and methods of processing.

Based on these results, the established method could be used to explain the chemical differentiation between raw XF samples and their different processed products, and to further understand the processing mechanism of herbal medicines for clinical practice. Traditional processing experience and modern science-technology were combined easily for quality control and the standardization of CHM processing.

## Figures and Tables

**Table 1:** Linear range, regression equation, R^2^, and limit of detection (LoD) precision of eight components.

Peak	Compound	Linear Regression					Precision Relative Standard Deviation (RSD) (%)		Recovery (%)	
		Regressive Equation ^1^	Linear Range (μg/mL)	R^2^	LOD (μg/mL)	LOQ (μg/mL)	Intra-Day (*n* = 6)	Inter-Day (*n* = 3)	Raw XF (RXF)	Processed XF (PXF)
2	5-CQA	*Y* = 23991*X* + 3544.6	1.31~131	0.9996	0.03	0.07	0.23	1.98	95.45~104.34	96.34~101.72
3	3-CQA	*Y* = 31325*X* − 106752	10.80~1080	0.9995	0.20	0.68	0.12	1.80	97.45~108.45	94.23~101.34
4	4-CQA	*Y* = 322269*X* − 54947	10.30~215	0.9995	0.17	0.58	1.62	1.97	96.22~103.67	95.67~105.21
5	CA	*Y* = 30259*X* − 54947	1.89~224	0.9998	0.13	0.45	1.03	1.53	94.78~102.77	95.69~107.23
6	1,3-diCQA	*Y* = 40113*X* − 83400	1.05~720	0.9991	0.06	0.21	1.57	1.88	99.76~105.38	98.23~104.09
9	1,5-diCQA	*Y* = 23487*X* − 310575	4.99~543	0.9995	0.02	0.07	1.75	2.07	95.46~100.98	94.67~100.45
11	1,3,5-tirCQA	*Y* = 19547*X* − 1918.3	2.34~642	0.9994	0.01	0.04	1.63	2.11	96.24~106.77	97.86~107.33
12	4,5-diCQA	*Y* = 33218*X* − 60842	1.55~234	0.9997	0.02	0.07	1.51	1.97	94.66~103.22	95.78~105.44

^1^ y and x refer to the peak area and the concentration of the analyte (μg/mL), respectively.

**Table 2:** Content of eight compounds in RXF (R1~R14).

No.	Region and Location (Latitude, Longitude)	Time of Collection (Specimen No.)	Content of Investigated Components (n = 3, mg/g ± SD)								Total Content (n = 3, mg/g ± SD)
			CA (5) ^1^	3-CQA (3)	4-CQA (4)	5-CQA (2)	1,3-diCQA (6)	1,5-diCQA (9)	1,3,5-tirCQA (11)	4,5-diCQA (12)	
R1	Binxian, Heilongjiang (45°45 N 127°28 E)	September, 2016 (RBH201609-01)	0.304 ± 0.010	7.080 ± 0.021	0.522 ± 0.009	0.094 ± 0.004	0.035 ± 0.003	1.250 ± 0.012	0.202 ± 0.009	0.158 ± 0 .003	9.645 ± 0.011
R2	Tieling, Lioaning (42°13 N 123°50 E)	September, 2016 (RTL201609-02)	0.168 ± 0.012	6.218 ± 0.022	0.502 ± 0.012	0.070 ± 0.011	0.089 ± 0.002	0.567 ± 0.009	0.346 ± 0.010	0.110 ± 0.004	8.070 ± 0.110
R3	Baotou, Inner Mongolia (40°39 N 109°50 E)	September, 2016 (RBI201609-03)	0.278 ± 0.013	6.800 ± 0.020	0.340 ± 0.013	0.088 ± 0.008	0.383 ± 0.004	3.512 ± 0.010	0.880 ± 0.008	0.274 ± 0.005	12.555 ± 0.123 *
R4	Baoding, Hebei (38°52 N 115°27 E)	October, 2016 (RBH201610-04)	0.288 ± 0.011	6.114 ± 0.022	0.364 ± 0.011	0.070 ± 0.013	0.048 ± 0.001	0.990 ± 0.006	0.677 ± 0.010	0.220 ± 0.003	8.771 ± 0.015
R5	Jining, Shandong (35°24 N 116°34 E)	September, 2016 (RJS201609-05)	0.176 ± 0.010	5.804 ± 0.019	0.604 ± 0.012	0.202 ± 0.011	0.042 ± 0.003	3.214 ± 0.011	0.258 ± 0.009	0.101 ± 0.003	10.401 ± 0.009
R6	Hanzhong, Shanxi (33°04 N 107°01 E)	September, 2016 (RHS201609-06)	0.288 ± 0.009	7.634 ± 0.020	0.676 ± 0.011	0.094 ± 0.012	0.128 ± 0.004	0.378 ± 0.007	0.196 ± 0.007	0.114 ± 0.004	9.508 ± 0.010
R7	Zhumadian, Henan (33°00 N 114°01 E)	October, 2016 (RZH201610-07)	0.418 ± 0.008	9.396 ± 0.020	0.892 ± 0.010	0.121 ± 0.010	0.669 ± 0.002	1.059 ± 0.009	0.238 ± 0.006	0.474 ± 0.003	13.267 ± 0.008 *
R8	Suqian, Jiangsu (33°57 N 118°16 E)	October, 2016 (RSJ201610-08)	0.378 ± 0.009	7.570 ± 0.023	0.758 ± 0.010	0.050 ± 0.011	0.068 ± 0.005	0.186 ± 0.008	0.038 ± 0.006	0.225 ± 0.006	9.273 ± 0.010
R9	Liuan, Anhui (31°44 N 116°31 E)	September, 2016 (RLA201609-09)	0.262 ± 0.010	6.658 ± 0.024	0.582 ± 0.012	0.536 ± 0.012	0.068 ± 0.003	0.735 ± 0.009	0.360 ± 0.004	0.109 ± 0.003	9.310 ± 0.009
R10	Dazhou, Sichuan (31°12 N 107°27 E)	October, 2016 (RDS201610-10)	0.896 ± 0.015	10.65 ± 0.021	0.456 ± 0.016	0.590 ± 0.014	6.257 ± 0.014	3.098 ± 0.023	5.409 ± 0.019	1.702 ± 0.010	29.058 ± 0.018 **
R11	Huanggang, Hubei (30°27 N 114°52 E)	September, 2016 (RHH201609-11)	0.290 ± 0.011	6.678 ± 0.021	0.380 ± 0.018	0.070 ± 0.010	0.018 ± 0.003	0.870 ± 0.009	0.279 ± 0.003	0.226 ± 0.006	8.811 ± 0.010*
R12	Shaoyang, Hunan (27°14 N 111°27 E)	October, 2016 (RSH201610-12)	0.670 ± 0.010	9.396 ± 0.013	0.596 ± 0.012	0.340 ± 0.019	0.049 ± 0.007	1.667 ± 0.012	0.148 ± 0.011	0.608 ± 0.013	13.474 ± 0.013 *
R13	Guilin, Guangxi (25°16 N 110°17 E)	October, 2016 (RGG201610-13)	0.374 ± 0.016	7.616 ± 0.017	0.532 ± 0.013	0.108 ± 0.004	0.139 ± 0.002	2.811 ± 0.011	0.537 ± 0.009	0.301 ± 0.005	12.418 ± 0.010 *
R14	Dali, Yunnan (25°36 N 100°15 E)	October, 2016 (RDY201610-14)	0.602 ± 0.013	4.296 ± 0.019	0.264 ± 0.011	0.128 ± 0.006	0.092 ± 0.003	0.096 ± 0.003	0.203 ± 0.007	0.412 ± 0.009	6.093 ± 0.009 *

^1^ Corresponds to the peak number in Figure 2. Compared with R9 group, * *p* < 0.05, ** *p* < 0.01.

**Table 3:** Content of eight compounds in PXF (P1~P16).

No.	Region or Pharmacy (Specimen No.)	Content of Investigated Components (n = 3, mg/g ± SD)								Total Content (n = 3, mg/g ± SD)
		CA (5) ^1^	3-CQA (3)	4-CQA (4)	5-CQA (2)	1,3-diCQA (6)	1,5-diCQA (9)	1,3,5-tirCQA (11)	4,5-diCQA (12)	
P1	Binxian, Heilongjiang (PBH2016-01)	0.223 ± 0.020	4.123 ± 0.032	1.051 ± 0.022	0.882 ± 0.010	0.056 ± 0.002	0.207 ± 0.004	0.114 ± 0.003	0.332 ± 0.005	6.988 ± 0.012
P2	Tieling, Lioaning (PTL2016-02)	0.196 ± 0.013	3.670 ± 0.029	0.824 ± 0.018	0.531 ± 0.012	0.045 ±0.003	0.178 ± 0.007	0.098 ± 0.004	0.222 ± 0.009	5.764 ± 0.014 *
P3	Baotou, Inner Mongolia (PBI2016-03)	0.331 ± 0.010	5.306 ± 0.029	0.912 ± 0.024	0.586 ± 0.025	0.174 ± 0.009	1.492 ± 0.012	0.423 ± 0.010	0.389 ± 0.014	9.613 ± 0.013
P4	Baoding, Hebei (PBH2016-04)	0.359 ± 0.012	5.024 ± 0.022	1.026 ± 0.021	0.891 ± 0.012	0.084 ± 0.010	0.273 ± 0.011	0.183 ± 0.012	0.330 ± 0.016	8.170 ± 0.012
P5	Jining, Shandong (PJS2016-05)	0.181 ± 0.015	3.940 ± 0.024	1.191 ± 0.022	1.120 ± 0.024	0.501 ± 0.014	0.501 ± 0.015	0.190 ± 0.009	0.656 ± 0.021	8.280 ± 0.015
P6	QInling, Shanxi (PQS2016-06)	0.121 ± 0.012	3.675 ± 0.022	0.630 ± 0.016	0.372 ± 0.011	0.097 ± 0.012	0.529 ± 0.014	0.079 ± 0.010	0.586 ± 0.014	6.089 ± 0.013*
P7	Zhumadian, Henan (PZH2016-07)	0.538 ± 0.014	7.238 ± 0.025	0.533 ± 0.012	0.140 ± 0.010	0.041 ± 0.011	1.068 ± 0.013	0.256 ± 0.012	0.317 ± 0.011	10.131 ± 0.014 *
P8	Suqian, Jiangsu (PSJ2016-08)	0.337 ± 0.010	5,142 ± 0.021	1.338 ± 0.011	1.145 ± 0.008	0.023 ± 0.009	0.017 ± 0.010	0.026 ± 0.011	0.714 ± 0.012	8.742 ± 0.013
P9	Liuan, Anhui (PLA2016-09)	0.042 ± 0.009	1.565 ± 0.012	0.428 ± 0.010	0.533 ± 0.012	0.079 ± 0.014	0.070 ± 0.012	0.085 ± 0.011	0.189 ± 0.012	2.991 ± 0.011 **
P10	Dazhou, Sichuan (PDS2016-10)	0.296 ± 0.015	7.567 ± 0.011	1.732 ± 0.012	1.221 ± 0.014	0.994 ± 0.011	9.688 ± 0.013	2.034 ± 0.16	2.677 ± 0.011	26.209 ± 0.012 **
P11	Huanggang, Hubei (PHH2016-11)	0.283 ± 0.013	3.306 ± 0.011	1.053 ± 0.012	0.871 ± 0.014	0.085 ± 0.011	0.143 ± 0.015	0.042 ± 0.013	0.246 ± 0.011	6.029 ± 0.011
P12	Shaoyang, Hunan (PSH2016-12)	0.161 ± 0.010	3.624 ± 0.012	0.768 ± 0.011	0.542 ± 0.013	0.084 ± 0.011	0.546 ± 0.014	0.129 ± 0.012	0.563 ± 0.013	6.417 ± 0.012
P13	Guilin, Guangxi (PGG2016-13)	0.209 ± 0.012	3.716 ± 0.011	0.901 ± 0.011	0.688 ± 0.023	0.067 ± 0.012	0.813 ± 0.021	0.137 ± 0.015	0.334 ± 0.020	6.865 ± 0.015
P14	Dali, Yunnan (PDY2016-14)	0.195 ± 0.015	2.983 ± 0.021	0.921 ± 0.016	0.853 ± 0.024	0.030 ± 0.009	0.976 ± 0.016	0.045 ± 0.008	0.166 ± 0.010	6.169 ± 0.014 *
P15	Hangzhou, Zhejiang (PHZ2016-15)	0.237 ± 0.021	5.607 ± 0.017	0.359 ± 0.012	0.338 ± 0.018	0.020 ± 0.004	0.670 ± 0.012	0.791 ± 0.021	0.469 ± 0.023	8.491 ± 0.016
P16	Tongrentang, Beijing (PTB2016-16)	0.406 ± 0.021	7.225 ± 0.016	0.697 ± 0.021	0.089 ± 0.010	0.103 ± 0.005	0.853 ± 0.011	0.232 ± 0.019	0.302 ± 0.021	9.907 ± 0.015

**^1^** Corresponds to the peak number in Figure 2. Compared with P16 group, * *p* < 0.05, ** *p* < 0.01.

## Supplementary Materials

The following are available online, Figure S1.
