# Supplementary figures and images for: HPLC-PDA Combined with Chemometrics for Quantitation of Active Components and Quality Assessment of Raw and Processed Fruits of Xanthium strumarium L
Source: Molecules. 2018 Jan 25;23(2):243. doi: 10.3390/molecules23020243 (PMC6017294; doi:10.3390/molecules23020243)

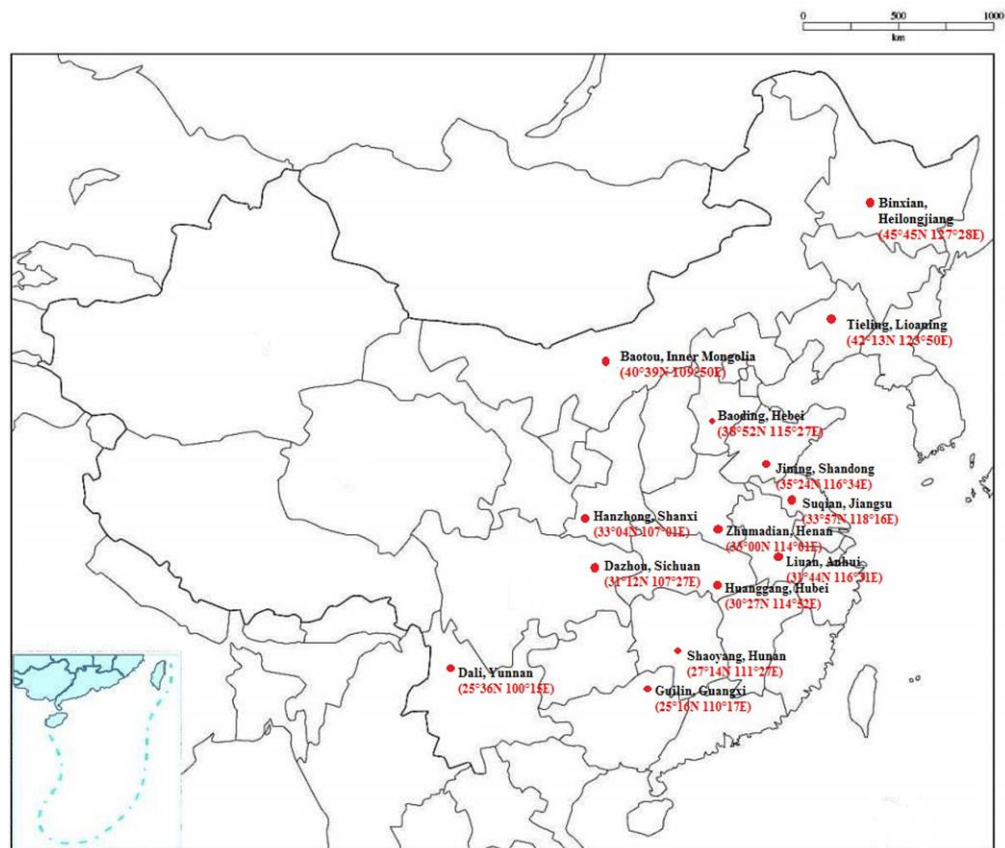

Figure S1. Map of region where the samples are collected (Red origin) in China.

Supplement: Supplementary file 1 [file molecules-23-00243-s001.pdf]
